# Point-SPV: end-to-end enhancement of object recognition in simulated prosthetic vision using synthetic viewing points

**DOI:** 10.3389/fnhum.2025.1549698

**Published:** 2025-03-24

**Authors:** Ashkan Nejad, Burcu Küçükoǧlu, Jaap de Ruyter van Steveninck, Sandra Bedrossian, Joost Heutink, Gera A. de Haan, Frans W. Cornelissen, Marcel van Gerven

**Affiliations:** ^1^Department of Research and Improvement of Care, Royal Dutch Visio, Huizen, Netherlands; ^2^Department of Machine Learning and Neural Computing, Donders Institute for Brain, Cognition and Behaviour, Radboud University, Nijmegen, Netherlands; ^3^Laboratory for Experimental Ophthalmology, University Medical Center Groningen, University of Groningen, Groningen, Netherlands; ^4^Faculty of Science and Engineering, University of Groningen, Groningen, Netherlands; ^5^Department of Clinical and Developmental Neuropsychology, University of Groningen, Groningen, Netherlands

**Keywords:** simulated prosthetic vision, synthetic viewing points, object recognition, end-to-end training, deep learning

## Abstract

Prosthetic vision systems aim to restore functional sight for visually impaired individuals by replicating visual perception by inducing phosphenes through electrical stimulation in the visual cortex, yet there remain challenges in visual representation strategies such as including gaze information and task-dependent optimization. In this paper, we introduce Point-SPV, an end-to-end deep learning model designed to enhance object recognition in simulated prosthetic vision. Point-SPV takes an initial step toward gaze-based optimization by simulating viewing points, representing potential gaze locations, and training the model on patches surrounding these points. Our approach prioritizes task-oriented representation, aligning visual outputs with object recognition needs. A behavioral gaze-contingent object discrimination experiment demonstrated that Point-SPV outperformed a conventional edge detection method, by facilitating observers to gain a higher recognition accuracy, faster reaction times, and a more efficient visual exploration. Our work highlights how task-specific optimization may enhance representations in prosthetic vision, offering a foundation for future exploration and application.

## 1 Introduction

Prosthetic vision holds great promise for restoring functional sight to individuals with visual impairments. Early research, such as the work by Brindley and Lewin (1968), demonstrated the potential for visual perception through electrical stimulation of the visual cortex, introducing the concept of “phosphenes” as the basic visual percept elicited by such stimulation. Since then, advancements in visual prosthetics have explored numerous avenues to enhance both the quality of perception and the functionality of devices. Researchers have worked on improving the perceived visual inputs, optimizing the representation, and addressing the challenge of providing naturalistic vision (Fernandez, 2018). One key area of focus is the incorporation of gaze information, which is crucial for a more intuitive and dynamic visual experience. Gaze integration is essential and gaze position significantly influences spatial localization (Paraskevoudi and Pezaris, 2019; Sabbah et al., 2014).

Even after loss of vision, people who had vision early in life still rely on the direction they move their eyes and head to update where they think things are in space (Reuschel et al., 2012). Consequently, any misalignment between the camera's orientation and the direction of the eyes causes the visual input to be perceived at an incorrect location. Traditional visual prosthetics often rely on head-steering mechanisms, which can result in disjointed or misaligned perceptions that hinder users in performing tasks such as object recognition, reading, or hand-eye coordination (Dagnelie et al., 2006; Titchener et al., 2018). This misalignment is caused by the lack of gaze compensation, as demonstrated in studies where prosthetic implant users experienced impaired spatial localization without integration of eye movements (Caspi et al., 2018; Sabbah et al., 2014).

Moreover, it has been shown that prosthetic vision without gaze compensation may lead to poorer performance in tasks requiring visual stability, such as object detection and reading (Paraskevoudi and Pezaris, 2021). This makes the inclusion of gaze information a crucial factor in enhancing object recognition and perceptual accuracy. De Ruyter van Steveninck et al. (2024) conducted a behavioral experiment with a virtual reality headset that showed that gaze-contingent representation in prosthetics led to improved speed and perceived quality of vision in tasks such as daily mobility, scene recognition, and visual search.

Research has shown that the human visual system exhibits task-dependent characteristics, processing visual information in a way that aligns with the specific objective or task at hand (Orban et al., 1996; Ballard and Hayhoe, 2009). Similarly, in simulated prosthetic vision (SPV), a representation method, the technique by which visual input from the camera is converted into a visual pattern or signal presented to the user, could benefit from a task-dependent approach. Such an approach would require designing and optimizing to address specific tasks, such as navigation or object recognition, thereby enabling an enhanced performance in targeted applications.

Vergnieux et al. (2017) demonstrated that simplifying visual renderings in simulated prosthetic vision could significantly improve navigation tasks, indicating the value of minimal yet informative visual cues. De Ruyter van Steveninck et al. (2022) utilized neural networks to optimize phosphene representations, focusing on a general-purpose visual enhancement. In contrast, Küçükoǧlu et al. (2022) took a task-dependent approach, using reinforcement learning to produce visual representations specifically for goal-directed mobility tasks. However, while these works provide frameworks for improving artificial vision, none of these takes gaze information into account.

To address the challenges of task-specific and gaze-informed optimization in prosthetic visual representations, we developed Point-SPV, an end-to-end deep learning model designed to enhance object representation in simulated prosthetic vision. This approach takes an initial step toward gaze-informed optimization by training the model on image patches extracted from around simulated viewing points. The viewing points can be considered to represent potential gaze locations, thereby improving the model's ability to adapt to gaze-specific visual information.

To test our approach, we conducted a screen-based gaze-contingent behavioral experiment comparing Point-SPV to a conventional edge-detection method, Canny edge detector (Canny, 1986). Edge detection has often been used as a standard in prosthetic vision simulations (De Ruyter van Steveninck et al., 2022; Vergnieux et al., 2017; Zhao et al., 2010), providing a baseline for visual information processing. In this experiment, participants were asked to discriminate between stimuli featuring cats and dogs. The performance of the participants was assessed in terms of discrimination accuracy, visual exploration, and reaction time. This approach allows us to investigate the task-specific representation efficiency of a model trained on simulated viewing points.

In summary, we propose the Point-SPV framework, which addresses task-specific representations in simulated prosthetic vision by utilizing simulated viewing points, serving as an initial step toward the incorporation of actual gaze information. In a behavioral experiment, we evaluate how our optimized model enhances object discrimination, exploring its potential to provide more effective object representations for individuals with visual impairments.

## 2 Point-SPV model

**Figure 1:**
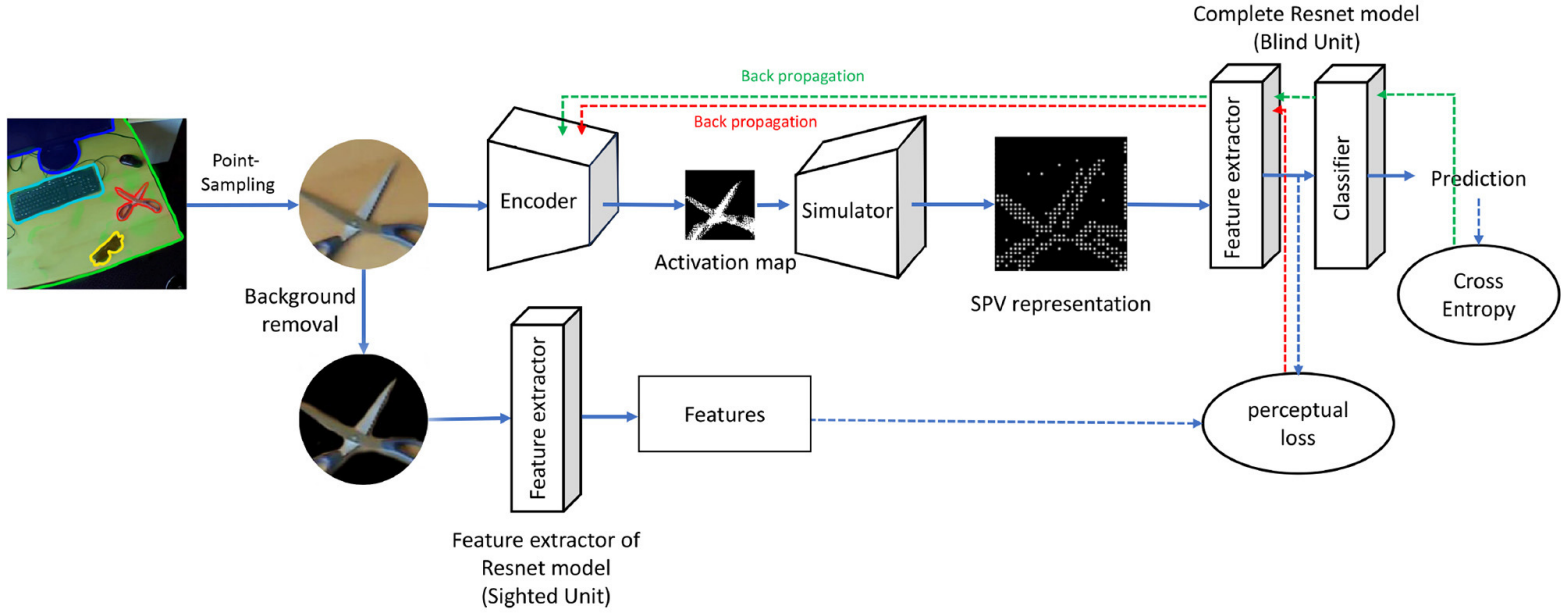
The pipeline begins with an input image, from which a viewing patch is extracted using a point sampling strategy (Section 2.1). A random viewing location is selected, and a patch around this point is cropped and fed into the encoder (Section 2.2). The encoder generates an activation map, which the simulator (Section 2.3) transforms into a simulated phosphene representation. This representation is passed to the blind unit (Section 2.4) for object recognition. Perceptual loss, calculated using feature representations from the sighted unit (Section 2.5), and cross-entropy loss for ground-truth categories are used to optimize trainable components, as indicated by the dashed lines.

This section outlines our end-to-end Point-SPV model, which consists of four primary components: an encoder, a simulator, a “blind” unit, and a “sighted” unit. Figure 1 provides an overview of the model pipeline, illustrating the flow of data and optimization process.

The training workflow begins with the extraction of a viewing patch from an input image using a point sampling strategy (Section 2.1). This patch is processed by the encoder (Section 2.2), which produces an activation map corresponding to electrode activations. The simulator (Section 2.3) converts this activation map into a simulated phosphene representation. The blind unit (Section 2.4) uses the phosphene representation for object recognition, while the sighted unit (Section 2.5) offers perceptual guidance through feature comparisons. Optimization of the model is driven by a combination of perceptual loss and cross-entropy loss, as described in Section 2.6.

### 2.1 Point sampling

**Figure 2:**
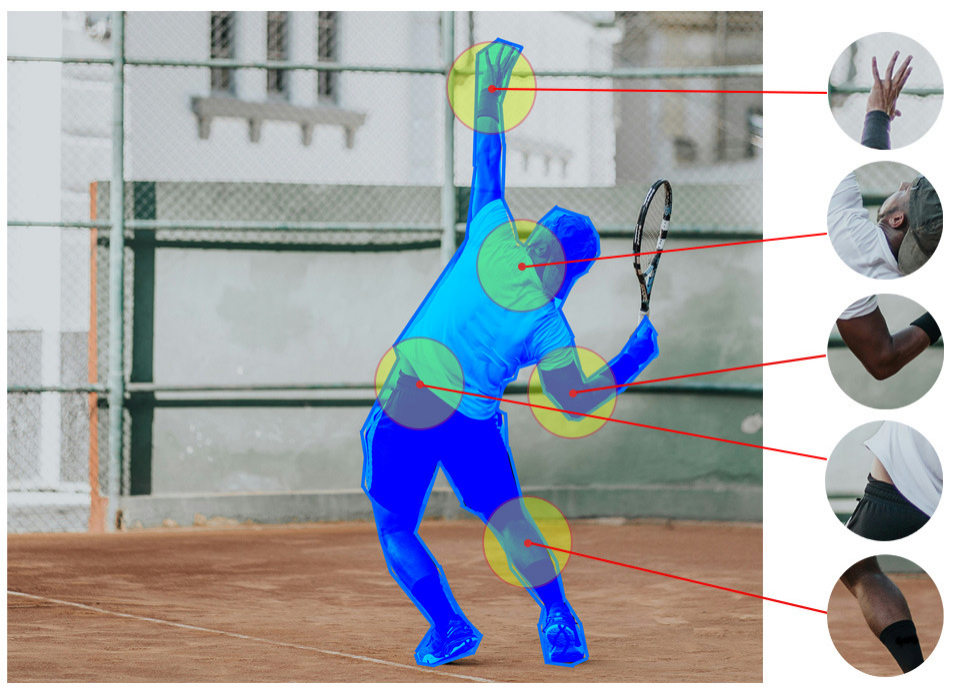
Random simulated viewing locations extracted from a target object based on the provided segmentation. The extracted patches are added either to the training or the validation set. The region with a blue overlay indicates the segmentation of the target object in the image while the yellow circles show the extracted viewing patches.

To train our model, we adopted a point sampling strategy to simulate a user's field of vision. We used the COCO dataset (Lin et al., 2014), which provides segmentation masks for target objects. The sampling process starts by identifying an object of interest within an image and applying its segmentation mask to isolate the object's boundaries. A random point within the masked area is selected as the possible viewing location, and a patch centered on this point is extracted. This patch represents the region the user would focus on during object recognition (Figure 2).

By adopting this approach, demonstrated in the Segment Anything method (Kirillov et al., 2023), we can strategically generate an abundance of training data by selecting multiple points on each object. Random point sampling has already proved to be advantageous, particularly when contrasted with utilizing segmentation from real human viewing recordings, as it circumvents the limitations and biases existing in human-centric data, such as annotation inconsistencies and selection bias. The deliberate randomness in our point sampling process contributes to a more robust and versatile model, capable of handling diverse scenarios.

### 2.2 Encoder

In this subsection, we discuss the architecture of our encoder which is responsible for transforming an input image of size 100 × 100 pixels into an activation map. The activation map represents the electrode activations on the implant array, corresponding to the input representation. Consistent with prior research (De Ruyter van Steveninck et al., 2022), we adopted a 32 × 32 array for the activation map.

The encoder is a fully convolutional deep neural network (DNN) architecture. Leaky rectified linear units are employed as the activation function in all layers except for the output layer, while batch normalization is integrated to ensure stable and efficient training. The detailed specification of the architecture is provided in Table 1. Contemporary cortical visual prostheses lack systematic control over phosphene brightness (Foroushani et al., 2018). Considering this limitation, and in line with the approach proposed by De Ruyter van Steveninck et al. (2022), we adopt a binary assumption for electrode activation in this study, eschewing graded activation. The output layer utilizes the Heaviside step function as its activation mechanism to produce binary stimulation values.

**Table 1:** Architecture of the encoder unit.

	**Type**	**In**	**Out**	**Size**	**Stride**	**Pad**	**Normalization**	**Activation**
1	Conv	3	8	3	1	1	BN	LReLU
2	Conv	8	16	3	1	1	BN	LReLU
3	Conv+Pool	16	32	3	1	1	BN	LReLU
4	Conv+Pool	32	64	3	1	1	BN	LReLU
5	Res	64/64	64/64	3/3	1/1	1/1	BN/BN	LReLU/LReLU
6	Res	64/64	64/64	3/3	1/1	1/1	BN/BN	LReLU/LReLU
7	Res	64/64	64/64	3/3	1/1	1/1	BN/BN	LReLU/LReLU
8	Res	64/64	64/64	3/3	1/1	1/1	BN/BN	LReLU/LReLU
9	Conv+Pool	64	32	3	1	1	BN	LReLU
10	Conv	32	16	3	1	1	BN	LReLU
11	Conv	16	8	3	1	1	BN	LReLU
12	Conv	8	3	3	1	1	-	LReLU
13	Conv	3	1	3	1	1	-	Sigmoid

Conv is convolutional layer; Res is residual block; Pool is max-pooling layer; BN is batch normalization; LReLU is leaky rectified linear unit.

### 2.3 Simulator

The second module of our model is the phosphene simulator, which transforms the stimulation pattern produced by the encoder into a simulated prosthetic vision representation. This module does not include trainable parameters and instead relies on predefined specifications to replicate the perceptual effects of electrical stimulation in visual prosthetics.

In the recent literature, various simulators have been introduced with the objective of achieving a more biologically plausible representation of visual stimuli through activation patterns (van der Grinten et al., 2024; Srivastava et al., 2009, 2007). For this study, we adopted a fundamental simulation of cortical prosthetic vision to demonstrate the training process of our end-to-end model. This simulation employs homogeneously distributed, equally-sized phosphenes, as outlined by De Ruyter van Steveninck et al. (2022). The choice of simulator is a simplified assumptions to lay the groundwork for more biologically plausible approaches, with a focus on incorporating gaze information in encoding strategies. We opted for binary patterns rather than biologically inspired phosphene generation methods to obtain a sound estimate of the impact of the model and its training process on phosphene informativeness.

In this approach, each element in the 32 × 32 stimulation protocol is mapped to preassigned pixels in a 256 × 256 image, creating the simulated visual field. Each element is rendered as a dot-shaped phosphene at the corresponding location in the output, effectively generating the simulated prosthetic vision representation.

### 2.4 Blind unit

The blind unit functions as a surrogate user, responsible for observing the simulation and identifying objects within the image. It uses the ResNet152 (He et al., 2016) classification model pretrained on the ImageNet dataset (Deng et al., 2009).

**Figure 3:**
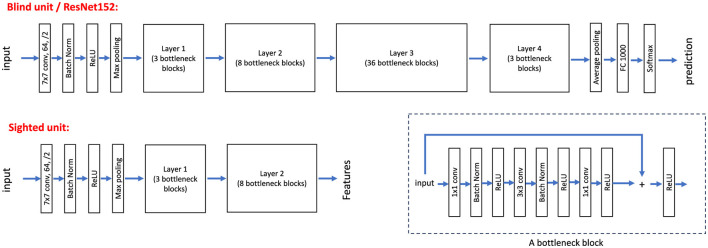
The blind unit is a full ResNet152 model, with the first six layers/units serving as the feature extractor and the remaining layers functioning as the classifier. For the sighted unit, only the first six layers/units of ResNet152 are used, similarly responsible for feature extraction.

In this study, we utilized the first six layers/units of ResNet152 for the feature extraction component and allocated the remaining layers for the classification section of the blind unit. This division was based on the observation that the shallower layers of the network retain low-level image features closely aligned with the original input, enabling both the blind and sighted units to converge toward a higher-level understanding of the input image. In contrast, deeper layers in deep neural networks extract more abstract and complex features, often discarding spatial information as typically perceived by humans (Erhan et al., 2009). The decision to divide ResNet152 at this specific layer was determined empirically. Figure 3 illustrates the architecture of ResNet152 as applied to our blind unit.

### 2.5 Sighted unit

The sighted unit utilizes the first six layers/units of the pretrained ResNet152 on the ImageNet dataset, which remain fixed during training. Similar to the blind unit, these initial layers are tasked with feature extraction, providing an abstract yet spatially coherent representation of the input image. Figure 3 illustrates the architecture of ResNet152 and the layers employed for the sighted unit in our pipeline.

The primary goal of the sighted unit is to align the blind unit's interpretation of visual information in simulated vision with the sighted unit's perception of the original image. This alignment is achieved through the application of perceptual loss, as detailed in Section 2.6. To focus on the essential aspects of the input, the sighted unit processes the target object without background, thereby encouraging the encoder to encode information specific to the object of interest.

### 2.6 Loss functions and optimization

The Point-SPV model is trained using a combination of two loss functions: perceptual loss (*L*_*P*_) and cross-entropy loss (*L*_*CE*_). These losses play complementary roles in optimizing the trainable components of the model, as illustrated in Figure 1. Perceptual loss is formulated as


(1) $$\begin{array}{l} L_{P}=\frac{||S_{i}\left(I^{\prime }\right)-B_{i}\left(V\left(E\left(I\right)\right)\right){||}_{2}^{2}}{W_{i}H_{i}C_{i}} \end{array}$$

where *B*_*i*_ and *S*_*i*_ denote the *i*th layer output of the blind unit and the sighted unit, respectively. *E* is the encoder and *V* is the simulator. The input simulated viewing patch with and without background are *I* and *I*′, respectively. *W*_*i*_, *H*_*i*_, and *C*_*i*_ are the shapes of the output at the *i*th layer. Equation 1 aligns the features extracted by the blind unit with those from the sighted unit. The sighted unit processes the viewing patch of the target object without including the background, generating a feature representation focused on the object. Meanwhile, the blind unit processes the SPV representation generated by the simulator, based on the activation map provided by the encoder. The loss is calculated by comparing mid-layer features of the blind unit with corresponding features from the sighted unit, using a squared L2 norm.

The perceptual loss influences both the encoder and the feature extraction layers of the blind unit. For the encoder, it ensures that the SPV representation remains consistent with the visual structure of the original image and that the SPV output is perceptually meaningful. Simultaneously, it optimizes the feature extraction component of the blind unit to extract features from the phosphene representation that match those extracted by the sighted unit, enabling the blind unit to adapt to the SPV.

Cross-entropy loss is applied to all layers of the blind unit, which predicts the object's class based on the SPV representation. This loss optimizes classification by minimizing the difference between the predicted class probabilities and the ground-truth labels. Additionally, the gradients from *L*_*CE*_ propagate back through the encoder, enhancing the quality of its output for object representation purposes.

The encoder and the feature extraction layers of the blind unit are optimized using both *L*_*P*_ and *L*_*CE*_ in the form of a weighted combination, formulated as


(2) $$\begin{array}{l} L=\gamma L_{P}+\left(1-\gamma \right)L_{CE} \end{array}$$

where γ is a value between zero and one. In this study, we empirically set γ to 0.75.

After training the Point-SPV model, it was essential to evaluate its effectiveness in supporting object recognition in humans. To achieve this, we conducted a behavioral experiment designed to test the model's performance, providing insights into its real-world applicability.

## 3 Behavioral experiment

To assess the effectiveness of Point-SPV, we conducted a screen-based gaze-contingent experiment comparing participant performances under two conditions: using Point-SPV or the Canny edge detection algorithm (Canny, 1986). Such edge detection techniques are commonly employed as standalone representation methods (Vergnieux et al., 2017) or for evaluating encoders (De Ruyter van Steveninck et al., 2024). Prior to executing the experiment, we performed a pilot study, which is described in the Supplementary material.

The experiment evaluated object discriminability across the two methods in terms of accuracy, reaction time, and visual exploration. We evaluated visual exploration by measuring the number of saccades per stimulus and the proportion of the image area covered by fixations. Together, these metrics provided a comprehensive view of how effectively participants processed and interpreted stimuli under each representation method.

### 3.1 Methods and materials

#### 3.1.1 Participants

Data was collected from 20 adult participants (13 female; mean age: 30 years, SD: 6.3 years; age range: 22–53 years). All participants reported to be healthy and have (corrected to) normal vision. The study received approval from the Ethical Committee of the Faculty of Behavioral and Social Sciences at the University of Groningen.

#### 3.1.2 Equipment

**Figure 4:**
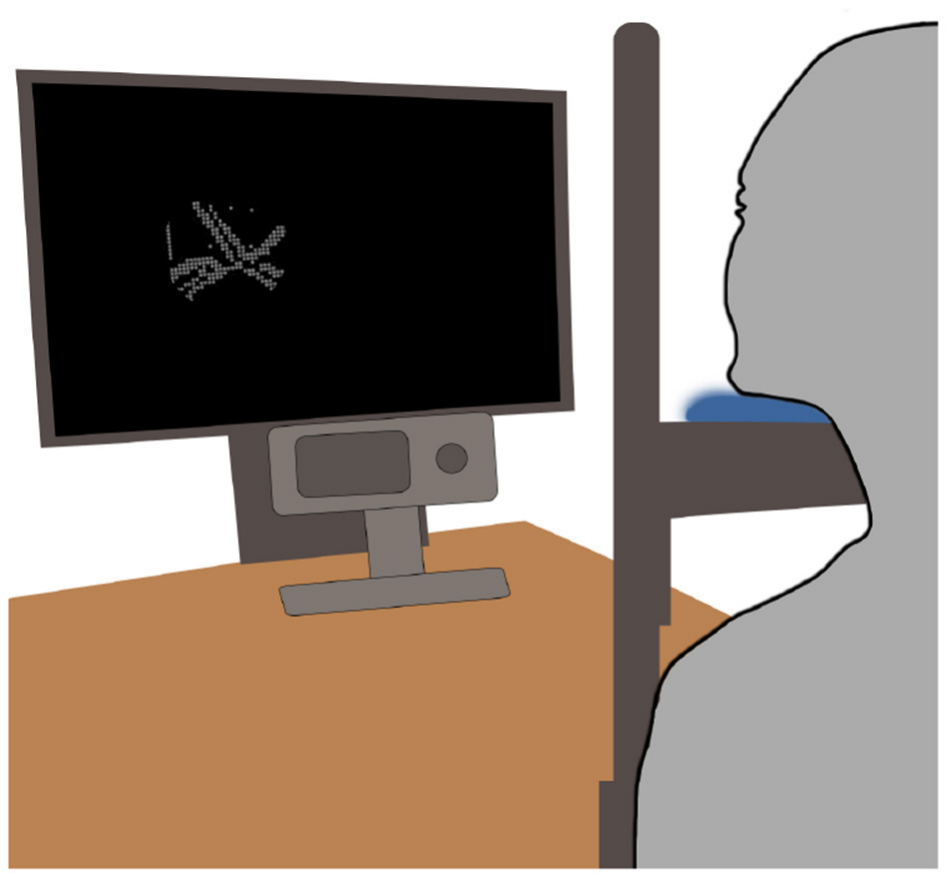
The experimental setup using a screen and a desk-mounted eye-tracker. The participant views the gaze-contingent stimuli on a screen while their eyes are being tracked via a desk-mounted eye-tracker.

The experiment utilized an EyeLink1000 eye-tracker with a chin-rest. The stimuli were displayed on a light-emitting diode (LED) backlight monitor (BenQ XL2540) with a 144 Hz refresh rate and a resolution of 1,920 × 1,080 pixels. The screen's luminance was measured at 52 cd/m^2^. The experimental setup is depicted in Figure 4. The stimuli were generated and displayed using a computer equipped with an Nvidia GTX 1060 3GB GPU and an Intel Core i7-8700 CPU. The behavioral experiment was implemented in Python using the PyLink library.

#### 3.1.3 Procedure and stimuli

The experimental design aimed for a direct comparison of the two methods while controlling for order effects and potential familiarity with the source images. For this reason, the participants were divided into two groups. For one group (10 participants), the first block featured stimuli processed using the Point-SPV method, while the second block presented the same stimuli processed using the Canny edge detection method. The other group (10 participants) performed the experiment in the reverse order, starting with the Canny edge filtering method in the first block and switching to Point-SPV in the second block.

**Figure 5:**
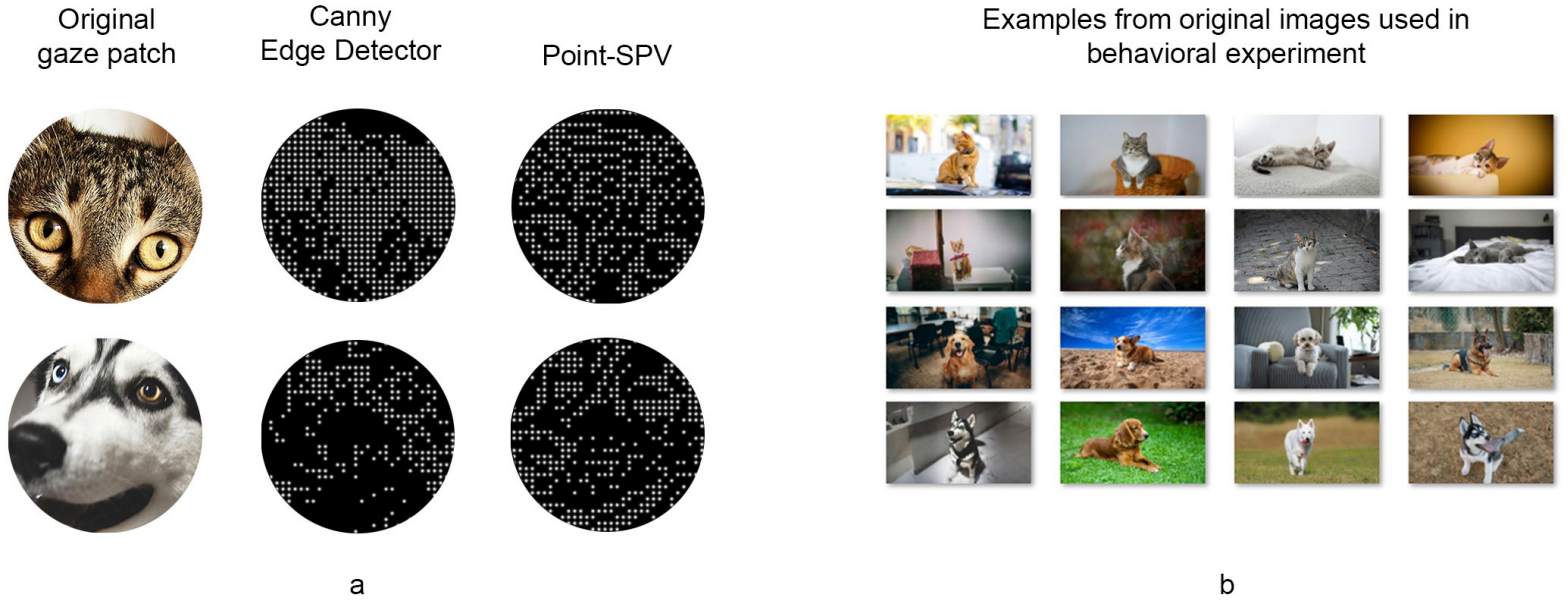
**(A)** Example outputs of the Canny edge detector and Point-SPV for two sample patches extracted from two images used in our behavioral experiment. **(B)** Examples of the original images presented via the two representation methods in our behavioral experiment.

Participants viewed images of cats and dogs placed against realistic backgrounds. These images were extracted from the internet and resized to the resolution of the screen. The stimuli were presented in a gaze-contingent manner, where only a circular region around the current viewing location was displayed using one of the representation methods. Example outputs of Point-SPV and Canny edge detection are shown in Figure 5A. The gaze-contingent setup captured momentary gaze location data, which was used to generate phosphene representations in real time. Each time the participant's gaze shifted, the system extracted a patch around the gaze location from the original image, processed it through the representation method, and displayed the result at the corresponding screen location. Figure 5B showcases examples of the original stimuli used in this behavioral experiment.

**Figure 6:**
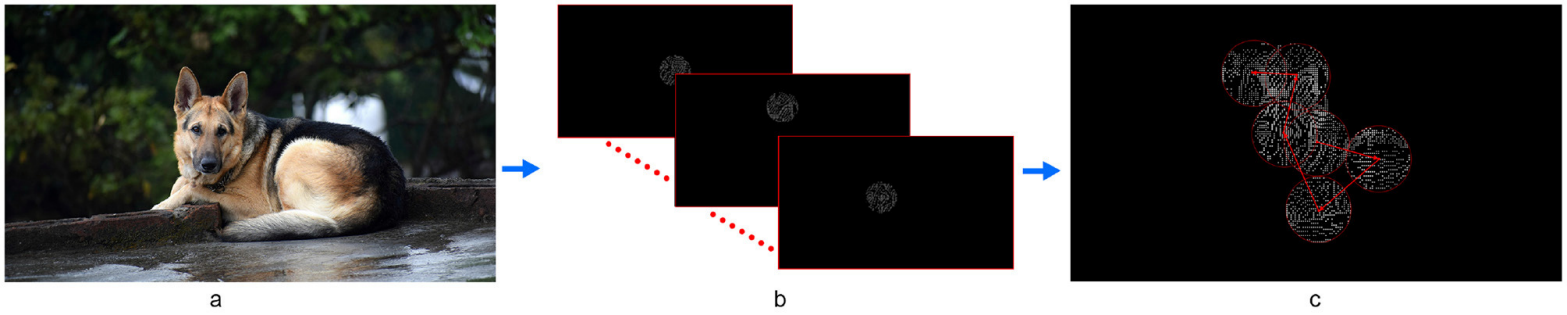
Illustration of the gaze-coupled phosphene representations used in the behavioral experiment. **(A)** A source image from our dataset used in the behavioral experiment. **(B)** An example of the scanned regions that provided visual cues for recognizing a target animal when presenting the original stimuli using Point-SPV in the gaze-contingent behavioral experiment. In **(B, C)**, each circle represents a fixation point during the trial, with the observed content displayed at that fixation. In **(C)**, the numbers indicate the sequential order of fixations, while the arrows depict the saccade trajectories between fixation points. Note that in the behavioral experiment, the phosphene representations were continuously updated, also during saccades. For clarity, here the representations during such transitions have been excluded from the illustration.

The gaze patches were substantially smaller than the target animals, thus requiring participants to scan the object to gather sufficient visual cues (e.g., the animal's leg, face, and ears) for complete recognition. Figure 6 shows a source image from the dataset overlaid with some examples of scanned regions with fixation points and saccade trajectories during the gaze-contingent behavioral experiment using Point-SPV. For clarity, we excluded the content that would have been presented during the saccades.

The experiment began with an eye-tracker calibration phase to ensure accurate eye-movement data. Eyelink's built-in 9-point calibration procedure was utilized to calibrate the eye movements before each block. Prior to each trial, participants fixated on a central green cross, guaranteeing that finding the target animal, which was always positioned at the center, did not require a search effort. Participants were thus informed about the target's location and were given up to 30 s to provide their response for each trial. During stimulus viewing, both eye movements and the final keypress response and the reaction time were recorded. Participants were instructed to indicate whether the stimulus contained a cat or a dog by pressing one of two designated buttons (left and right arrow keys) on the keyboard. We used the EyeLink1000 analysis software default settings for saccade detection.

Each participant completed two blocks of 50 trials each. The same set of images was used in both blocks. Within each block, the order of presentation was randomized. For each trial, we recorded the participant's keypress response (cat/dog decision) and reaction time. We also collected continuous eye-tracking data, from which we derived saccade events and viewing locations using the EyeLink1000 analysis software using default settings for saccade detection.

#### 3.1.4 Data analysis

Four primary performance metrics were computed: accuracy, reaction time, number of saccades, and stimulus coverage. Per block, these metrics were averaged across all stimuli presented to a participant.

Accuracy was measured as the proportion of correctly categorized stimuli, and reflects participants' ability to extract the relevant information for distinguishing the categories from the displayed phosphene representations. Reaction time refers to the elapsed time from stimulus onset to the participant's response, and reflects the time required for processing the represented information and making a decision.

Number of saccades was calculated by counting the number of saccades during each trial until the moment of the response. These rapid eye movements between fixation points provided insights into participants' visual exploratory behavior.

To compute stimulus coverage, we modeled each viewing point as a two-dimensional Gaussian distribution (σ = 50) centered at its location within the viewing patch. We then applied these Gaussian distributions over time and across the image, allowing them to overlap. At each pixel, we took the maximum Gaussian value from all overlapping distributions to form a continuous heatmap. To obtain a single coverage metric, we summed the Gaussian values across all pixels in the resulting heatmap and normalized this sum by the total number of pixels. This procedure produced a coverage metric that could range continuously up to 100%, where 100% ccoverage for one stimulus would indicate that every pixel in the image was encompassed by at least one viewing patch.

#### 3.1.5 Statistical analysis

Each participant viewed the same set of stimuli under both representation methods, and all performance metrics were analyzed by combining data from both conditions, regardless of the order in which the methods were presented. With this approach, we balanced out any potential learning or familiarity effects that might arise from the exposure to the original images. Subsequently, a repeated measures ANOVA test was conducted for each performance metric to evaluate differences between Point-SPV and Canny edge detection.

## 4 Results

In this section, participants' visual discrimination performance, for phosphene representations generated using either our Point-SPV or the Canny edge detection method, is compared in terms of their accuracy, reaction time, and visual exploration, with the latter assessed through number of saccades and stimulus coverage.

### 4.1 Accuracy

**Figure 7:**
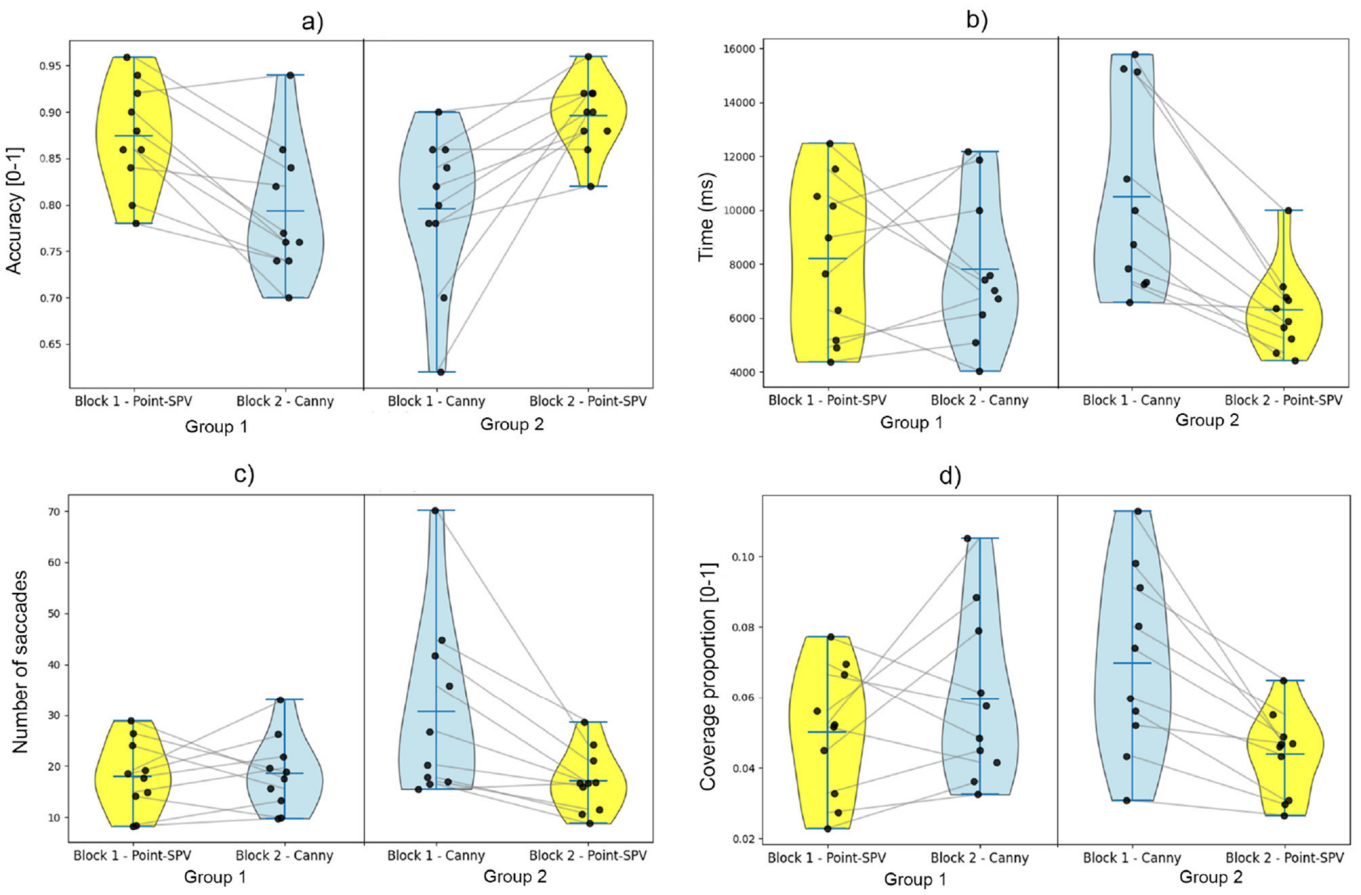
Comparison of behavioral metrics across blocks and methods (Point-SPV and Canny edge detection). Metrics include overall accuracy, reaction time, number of saccades, and stimulus coverage proportion, presented as violin plots with individual participant data points. Yellow-colored columns represent metrics obtained from participants using Point-SPV, and blue-colored columns correspond to the Canny edge detector. **(A)** Accuracy. **(B)** Reaction time. **(C)** Number of saccades. **(D)** Stimulus coverage.

As shown in Figure 7A, participants first viewing Point-SPV-generated representations achieved an average accuracy of 87% (*SD* = 5.8%), compared to 80% (*SD* = 8.3%) for those who first viewed edge detection generated representations. In the second block, among the participants who switched from Point-SPV to edge detection in the second block, 9 out of 10 showed a decline in accuracy, dropping to 80% (*SD* = 7.1%) on average. In contrast, the performance of the participants who started with edge detection and switched to Point-SPV demonstrated improved, with accuracy rising to 89% (*SD* = 3.8%) on average.

The results differed significantly between the two methods, with participants using Point-SPV achieving significantly higher accuracy [F_(1, 19)_ = 31.6, *p* < 0.001]. The average accuracy of the participants was approximately 88% (*SD* = 5.1%) when using Point-SPV, compared to 79% (*SD* = 7.9%) when using Canny edge detection.

### 4.2 Reaction time

As illustrated in Figure 7B, participants starting with Point-SPV had an average reaction time of about 8 s (*SD* = 2.9), while those beginning with edge detection responded in about 11 seconds on average (*SD* = 3.6). When participants switched from Point-SPV to edge detection, their reaction times remained similar. However, participants transitioning from edge detection to Point-SPV responded noticeably faster with an average reaction time of 6 s (*SD* = 1.6).

Participants had an average reaction time of approximately 6 s (*SD* = 2.15) with Point-SPV, compared to 8 s (*SD* = 3.53) with Canny edge detection. This difference between methods was significant [F_(1, 19)_ = 5.3, *p* = 0.03].

### 4.3 Number of saccades

As shown in Figure 7C, participants using Point-SPV in the first block made on average 18 saccades per trial (*SD* = 6.9), whereas those starting with edge detection showed higher numbers, averaging 31 saccades per trial (*SD* = 17.6). For participants who switched from Point-SPV to edge detection, the number of saccades remained relatively stable around 18 saccades (*SD* = 7.2). In contrast, those transitioning from edge detection to Point-SPV showed a reduction in the number of saccades, from 30 to 17 saccades (*SD* = 6.1).

Our findings from the paired samples t-test for the number of saccades were significant, demonstrating that participants using the Point-SPV method performed significantly less saccades [*F*_(1, 19)_ = 6.2, *p* = 0.02] than the edge detection method. Participants exhibited an average number of saccades of approximately 16 saccades (*SD* = 6.26) when using Point-SPV, compared to 24 saccades (*SD* = 15.06) with Canny edge detection.

### 4.4 Stimulus coverage

**Figure 8:**
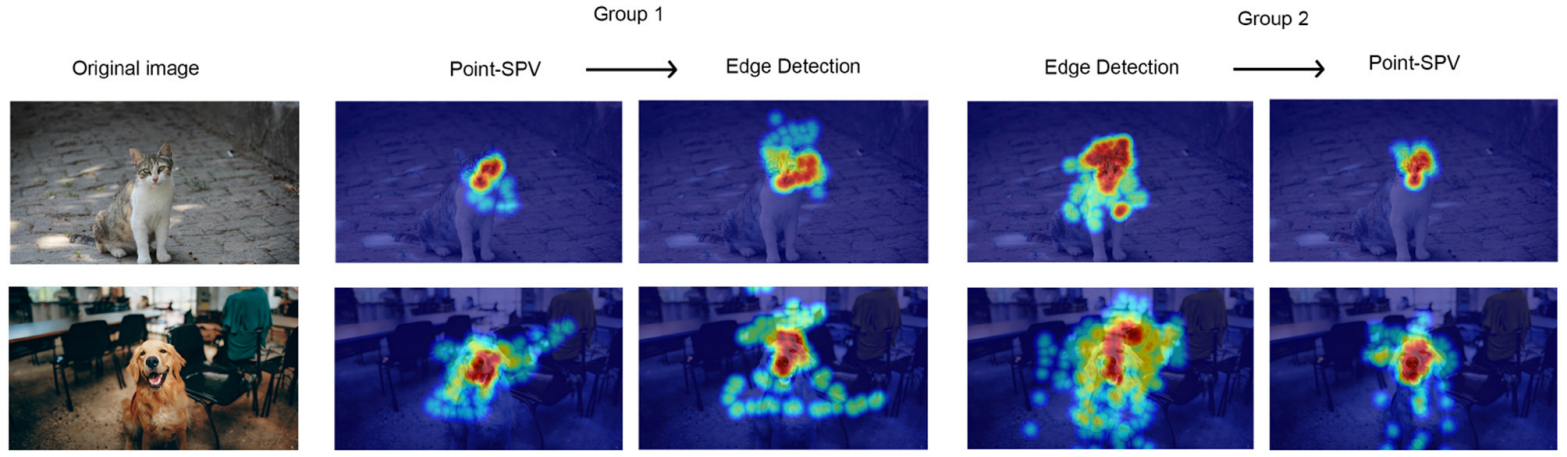
Average fixation heatmaps for two example stimuli across experimental conditions. Heatmaps are shown separately for Group 1 (switching from Point-SPV to edge detection) and Group 2 (switching from edge detection to Point-SPV), with visual attention averaged across all participants in each block. Warmer colors indicate areas with higher fixation density.

As shown in Figure 7D, stimulus coverage analysis revealed that participants using Point-SPV in the first block covered an average of 5% of the stimulus area (*SD* = 0.01), while those starting with edge detection had a higher coverage, around 7% (*SD* = 0.02). After switching representation methods, participants transitioning from Point-SPV to edge detection showed a slight increase in coverage, from 5% to 6%, whereas those moving from edge detection to Point-SPV exhibited a decrease (from 7% to 4%). Figure 8 shows the average fixated areas for two example stimuli, calculated separately for each block and averaged across all participants. On average, participants explored about 4% of the stimulus area (*SD* = 0.015) with Point-SPV, whereas the coverage increased to 6% (*SD* = 0.024) when using Canny edge detection. Thus, participants in the Point-SPV condition tended to inspect smaller regions of the stimuli [*F*_(1, 19)_ = 11.6, *p* = 0.002].

## 5 Discussion

In this study, we introduce Point-SPV, an end-to-end deep learning model for prosthetic vision that incorporates simulated viewing to enhance object recognition for observers. This work serves as a proof of concept for how simulated viewing points can be integrated into the optimization process of a as an initial step for gaze-based optimization of representation models. Our primary aim is to demonstrate the feasibility of incorporating gaze-inspired mechanisms in phosphene representations. In a behavioral experiment, we demonstrated that the representations generated by Point-SPV, in comparison to the ones generated by a conventional edge detection method, made it easier and faster for observers to distinguish between cats and dogs shown on realistic backgrounds. Moreover, when using Point-SPV, participants made fewer saccades and their gaze covered a smaller part of the stimuli, indicating our new model generates more efficient representations.

Moreover, our task-oriented approach is supported by the understanding that the human visual system processes information differently depending on the specific task (Orban et al., 1996). Point-SPV enhances the relevance of the visual information presented to the user by tailoring the representation method specifically to the object recognition task. This is accomplished through a task-specific architecture that includes an object classification unit (the blind unit), where the encoder is additionally optimized based on how effectively its representations can be recognized by the object classifier unit. This approach contrasts with previous methods that focused on general-purpose optimization without considering viewing information integration or task specificity (De Ruyter van Steveninck et al., 2022; Relic et al., 2022). However, previous methods could also benefit from incorporating aspects of the Point-SPV training process in their frameworks.

One of the key strengths of Point-SPV is its end-to-end deep learning design, which jointly optimizes both the encoding and final representation stages to better support human object recognition. By integrating the entire processing pipeline within a single trainable framework, the system is guided not only by classification objectives but also by perceptual cues derived from a sighted reference unit. This approach encourages the learned representations to be spatially consistent, perceptually meaningful, and directly useful to human observers.

The results of the behavioral experiment highlight the advantages of the Point-SPV representation method across various performance metrics. The approximately 9% overall gain in accuracy and the approximately 3 seconds reduction in response time demonstrated that Point-SPV's representations significantly enhanced participants' ability to correctly and more rapidly categorize stimuli, compared to those based on edge detection. It is important to note, that the observed performance gains cannot be solely attributed to the incorporation of gaze information, as other factors, such as the filtering methods, also play a role. This paper proposes an optimization process based on viewing points and our behavioral experiment highlights the potential in such an optimization strategy.

We also measured the number of saccades and the proportion of the stimulus covered. Participants using Point-SPV required fewer saccades and a smaller stimulus coverage, suggesting Point-SPV allowed for a more focused and efficient exploration of the stimuli.

Using our present hardware, Point-SPV was capable of presenting real-time computed representations of stimulus patches with a refresh rate of 60 Hz. This frequency ensured a sufficiently smooth and responsive user experience. Such real-time processing is essential for practical deployment in assistive devices, as latency can significantly impact usability and user comfort.

## 6 Limitations and future directions

For the training of our model, we utilized viewing patches, which represent a simplified version of natural viewing behavior. However, this approach does not account for the saccadic patterns characteristic of natural vision (Nejad et al., 2024; Kothari et al., 2020). Future research could enhance these models by incorporating natural gaze behavior, expanding the architecture to process a continuous stream of gaze positions. Our study introduced a proof-of-concept for including viewing points in the training process. Future work should explore integrating saccadic patterns and top-down gaze influences to increase validity.

Parameter choices, such as the γ value balancing perceptual and cross-entropy loss, the depth at which ResNet152 was divided into feature extractor and classifier, and the thresholds for Canny edge detection, were based on empirical observations to achieve the best performance. Future work can explore the effect of changes in the mentioned parameter. Future work can also focus on comparing our model to other end-to-end models, testing them using more biologically plausible simulators (van der Grinten et al., 2024) to investigate the shortcomings and advantages among all available methods. In our study, a simulator with homogeneously distributed and equally-sized phosphenes was chosen because this study serves as a proof-of-concept with simplified assumptions, laying the groundwork for future biologically plausible approaches. Our model is designed with flexibility, allowing the simulator to be replaced and the system to be retrained accordingly. Future work can also focus on utilizing and designing automatic quality assessment methods (Tolie et al., 2024) to assess the content of the representation prior to behavioral experiments.

The behavioral experiment was conducted in a controlled lab environment using a screen-based gaze-contingent display, which may not fully reflect real-world prosthetic vision use. The controlled setup isolated the impact of the representation method but lacked many aspects of dynamic, real-world tasks. This underscores the need for future research in virtual or augmented reality. To illustrate how our model could work in the context of a complex real-world multi-object environment, we applied it to a recording from a mobile eye-tracker while viewing objects. This video is provided in the Supplementary material.

Although our study balanced the order of representation methods across blocks, we did not specifically analyze potential order or interaction effects in the behavioral results. The presence or absence of such effects remains unconfirmed, and future studies could explore whether the sequence of methods affects the performance metrics.

Our pilot behavioral experiment, included in the Supplementary material, suggested that the effectiveness of Point-SPV depends on stimulus characteristics. In an animate-versus-inanimate task using images with white backgrounds, both methods performed similarly and near ceiling levels. However, this can suggest that Point-SPV's advantages could be influenced by the specific images or categories used.

## 7 Conclusion

Our proposed method, Point-SPV, represents an advancement in the field of prosthetic vision by integrating simulated viewing points and employing a task-oriented design to enhance object recognition. Our end-to-end deep learning model takes an initial step toward training of models using gaze information and proposes an optimization approach for object representation. Our behavioral findings illustrate the potential gain of our task-based optimization on simulated viewing patches. By tailoring the visual representation to the specific needs of the task and the user, Point-SPV offers a promising pathway toward more effective and naturalistic representation in prosthetic vision.

## Data Availability

The processed data supporting the conclusions of this article will be made available by the authors, without undue reservation.

## References

[B1] Ballard D. H. Hayhoe M. M. (2009). Modelling the role of task in the control of gaze. Vis. Cogn. 17, 1185–1204. 10.1080/13506280902978477 20411027 PMC2856937

[B2] Brindley G. S. Lewin W. S. (1968). The sensations produced by electrical stimulation of the visual cortex. J. Physiol. 196, 479–493. 10.1113/jphysiol.1968.sp008519 4871047 PMC1351724

[B3] Canny J. (1986). A computational approach to edge detection. IEEE Trans. Pattern Analy. Mach. Intellig. 8, 679–698. 10.1109/TPAMI.1986.4767851 21869365

[B4] Caspi A. Roy A. Wuyyuru V. Rosendall P. E. Harper J. W. Katyal K. D.. (2018). Eye movement control in the Argus II retinal-prosthesis enables reduced head movement and better localization precision. Investigat. Ophthalmol. Visual Sci. 59, 792–802. 10.1167/iovs.17-22377 29392324

[B5] Dagnelie G. Walter M. Yang L. (2006). Playing checkers: detection and eye-hand coordination in simulated prosthetic vision. J. Modern Optics 53, 1325–1342. 10.1080/09500340600619197

[B6] De Ruyter van Steveninck J. Güçlü U. van Wezel R. van Gerven M. (2022). End-to-end optimization of prosthetic vision. J. Vis. 22:2. 10.1167/jov.22.2.20 35703408 PMC8899855

[B7] De Ruyter van Steveninck J. Nipshagen M. van Gerven M. Güçlü U. Güçlüturk Y. van Wezel R. (2024). Gaze-contingent processing improves mobility, scene recognition and visual search in simulated head-steered prosthetic vision. J. Neural Eng. 21:026037. 10.1088/1741-2552/ad357d 38502957

[B8] Deng J. Dong W. Socher R. Li L.-J. Li K. Fei-Fei L. (2009). “Imagenet: a large-scale hierarchical image database,” in 2009 IEEE Conference on Computer Vision and Pattern Recognition (Miami, FL: IEEE), 248–255.

[B9] Erhan D. Bengio Y. Courville A. Vincent P. (2009). Visualizing Higher-Layer Features of a Deep Network. Montreal, QC: University of Montreal, 1.

[B10] Fernandez E. (2018). Development of visual neuroprostheses: trends and challenges. Bioelect. Med. 4:12. 10.1186/s42234-018-0013-8 32232088 PMC7098238

[B11] Foroushani A. N. Pack C. C. Sawan M. (2018). Cortical visual prostheses: from microstimulation to functional percept. J. Neural Eng. 15:021005. 10.1088/1741-2552/aaa904 29350199

[B12] He K. Zhang X. Ren S. Sun J. (2016). “Deep residual learning for image recognition,” in Proceedings of the IEEE Conference on Computer Vision and Pattern Recognition (Las Vegas, NV: IEEE), 770–778.

[B13] Kirillov A. Mintun E. Ravi N. Mao H. Rolland C. Gustafson L.. (2023). Segment anything. arXiv [preprint] arXiv:2304.02643. 10.1109/ICCV51070.2023.00371

[B14] Kothari R. Yang Z. Kanan C. Bailey R. Pelz J. B. Diaz G. J. (2020). Gaze-in-wild: A dataset for studying eye and head coordination in everyday activities. Sci. Rep. 10:2539. 10.1038/s41598-020-59251-5 32054884 PMC7018838

[B15] Küçükoǧlu B. Rueckauer B. Ahmad N. de Ruyter van Steveninck J. Güçlü U. van Gerven M. (2022). Optimization of neuroprosthetic vision via end-to-end deep reinforcement learning. Int. J. Neural Syst. 32:2250052. 10.1142/S0129065722500526 36328967

[B16] Lin T. Maire M. Belongie S. J. Bourdev L. D. Girshick R. B. Hays J.. (2014). Microsoft COCO: common objects in context. arXiv [preprint] arXiv:1405.0312. 10.1007/978-3-319-10602-1_48

[B17] Nejad A. de Haan G. A. Heutink J. Cornelissen F. W. (2024). ACE-DNV: Automatic classification of gaze events in dynamic natural viewing. Behavior Res. Methods 2024, 1–15. 10.3758/s13428-024-02358-8 38448726 PMC11133063

[B18] Orban G. A. Dupont P. Vogels R. De Bruyn B. Bormans G. Mortelmans L. (1996). Task dependency of visual processing in the human visual system. Behav. Brain Res. 76, 215–223. 10.1016/0166-4328(95)00195-6 8734055

[B19] Paraskevoudi N. Pezaris J. S. (2019). Eye movement compensation and spatial updating in visual prosthetics: mechanisms, limitations and future directions. Front. Syst. Neurosci. 12:73. 10.3389/fnsys.2018.00073 30774585 PMC6368147

[B20] Paraskevoudi N. Pezaris J. S. (2021). Full gaze contingency provides better reading performance than head steering alone in a simulation of prosthetic vision. Sci. Rep. 11:1. 10.1038/s41598-021-86996-4 34045485 PMC8160142

[B21] Relic L. Zhang B. Tuan Y.-L. Beyeler M. (2022). “Deep learning-based perceptual stimulus encoder for bionic vision,” in Proceedings of the Augmented Humans International Conference 2022, 323–325.

[B22] Reuschel J. Rösler F. Henriques D. Y. Fiehler K. (2012). Spatial updating depends on gaze direction even after loss of vision. J. Neurosci. 32, 2422–2429. 10.1523/JNEUROSCI.2714-11.2012 22396416 PMC6621798

[B23] Sabbah N. Authié C. N. Sanda N. Mohand-Said S. Sahel J. A. Safran A. B. (2014). Importance of eye position on spatial localization in blind subjects wearing an argus II retinal prosthesis. Investigat. Ophthalmol. Visual Sci. 55, 8259–8266. 10.1167/iovs.14-15392 25414187

[B24] Srivastava N. Troyk P. Towle V. Curry D. Schmidt E. Kufta C.. (2007). “Estimating phosphene maps for psychophysical experiments used in testing a cortical visual prosthesis device,” in 2007 3rd International IEEE/EMBS Conference on Neural Engineering (Kohala Coast, HI: IEEE), 130–133.

[B25] Srivastava N. R. Troyk P. R. Dagnelie G. (2009). Detection, eye-hand coordination and virtual mobility performance in simulated vision for a cortical visual prosthesis device. J. Neural Eng. 6:035008. 10.1088/1741-2560/6/3/035008 19458397 PMC3902177

[B26] Titchener S. A. Shivdasani M. N. Fallon J. B. Petoe M. A. (2018). Gaze compensation as a technique for improving hand–eye coordination in prosthetic vision. Transl. Vision Sci. Technol. 7:2. 10.1167/tvst.7.1.2 29321945 PMC5759363

[B27] Tolie H. F. Faraji M. R. Qi X. (2024). Blind quality assessment of screen content images via edge histogram descriptor and statistical moments. Vis. Comput. 40, 5341–5356. 10.1007/s00371-023-03108-1

[B28] van der Grinten M. van Steveninck J. R. Lozano A. Pijnacker L. Rueckauer B. Roelfsema P.. (2024). Towards biologically plausible phosphene simulation for the differentiable optimization of visual cortical prostheses. Elife 13:e85812. 10.7554/eLife.85812 38386406 PMC10883675

[B29] Vergnieux V. Macé M. J.-M. Jouffrais C. (2017). Simplification of visual rendering in simulated prosthetic vision facilitates navigation. Artif. Organs 41, 852–861. 10.1111/aor.12868 28321887

[B30] Zhao Y. Lu Y. Tian Y. Li L. Ren Q. Chai X. (2010). Image processing based recognition of images with a limited number of pixels using simulated prosthetic vision. Inf. Sci. 180, 2915–2924. 10.1016/j.ins.2010.04.021

