# Supplementary material for: Point-SPV: end-to-end enhancement of object recognition in simulated prosthetic vision using synthetic viewing points
Source: Front Hum Neurosci. 2025 Mar 24;19:1549698. doi: 10.3389/fnhum.2025.1549698 (PMC11973266; doi:10.3389/fnhum.2025.1549698)
Supplement: Supplementary file 1 [file Data_Sheet_1.pdf]

Supplementary Material

In this section, we present supplementary results and analyses that provide additional context to our study. Before conducting the behavioral experiment described in the main manuscript, we performed a pilot study using a different categorization task. Additionally, we describe the intermediate pipeline configurations tested during the development of Point-SPV, which contributed to shaping the final architecture. Finally, we include an overview of the changes in loss values for the training and validation sets, as well as the validation accuracy, observed during the model's training process.

1 PILOT STUDY

In our pilot experiment, we presented 50 images of animals and inanimate objects randomly using the two representation methods. Nineteen participants (distinct from those in the final experiment) were asked to distinguish between the two categories using a button response. The image set consisted of an equal number of both categories, and all participants viewed the same set of stimuli in random order. In this experiment, the original images had white backgrounds. Figure S1b illustrates examples of stimuli used in the pilot experiment. Figure S1a demonstrates example outputs of our method and Canny edge detector for two example viewing patches.

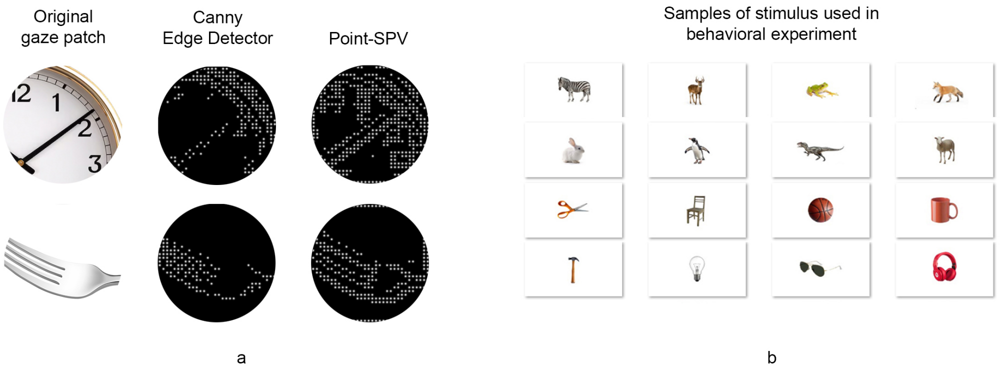

Figure S1. a) Example outputs of the Canny edge detector and Point-SPV for two sample patches extracted from two images used in our behavioral experiment. b) Examples from the original images presented via representation methods in our behavioral experiment.

1.1 Accuracy

Both Point-SPV and Canny edge detection demonstrated high levels of accuracy, suggesting a potential ceiling effect. Participants who started with Point-SPV in the first block achieved an average accuracy of approximately 94%, while those beginning with edge detection performed similarly well, with an average accuracy of about 92%. When participants switched from Point-SPV to edge detection in the second block, their accuracy remained relatively stable, showing minimal change and further supporting the notion that both methods performed similarly well under these conditions. Similarly, participants who started with edge detection and then transitioned to Point-SPV maintained consistently high accuracy levels.

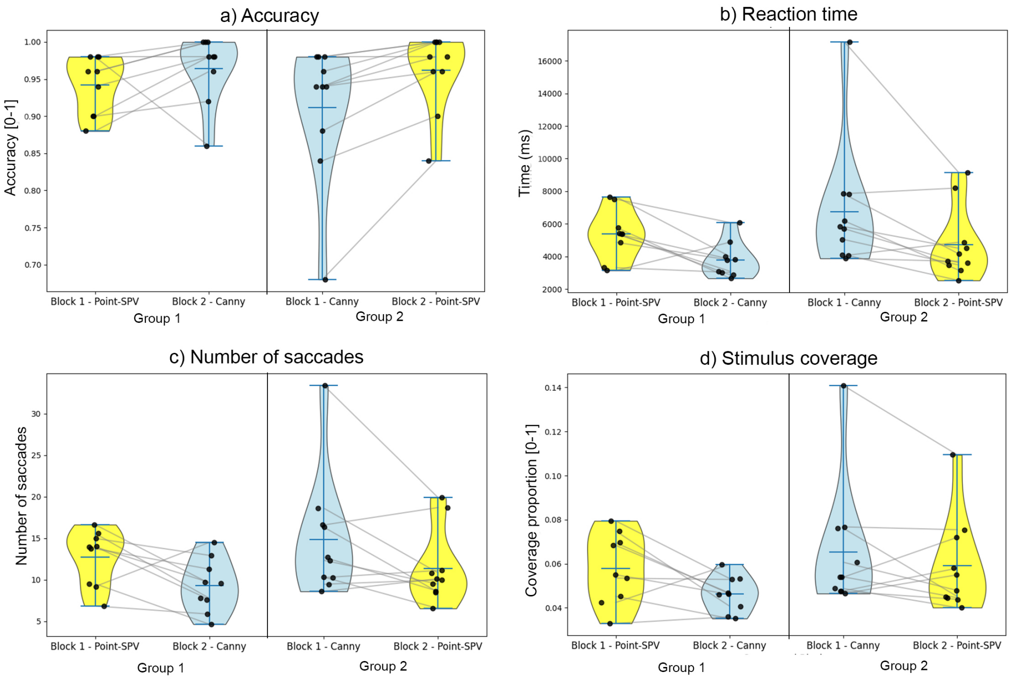

Figure S2. Comparison of behavioral metrics across blocks and methods (Point-SPV and Canny edge detector). Metrics include overall accuracy, reaction time, number of saccades, and stimulus coverage proportion, presented as violin plots with individual participant data points. Yellow-colored columns represent metrics obtained from participants using Point-SPV, and blue-colored columns correspond to the Canny edge detector.

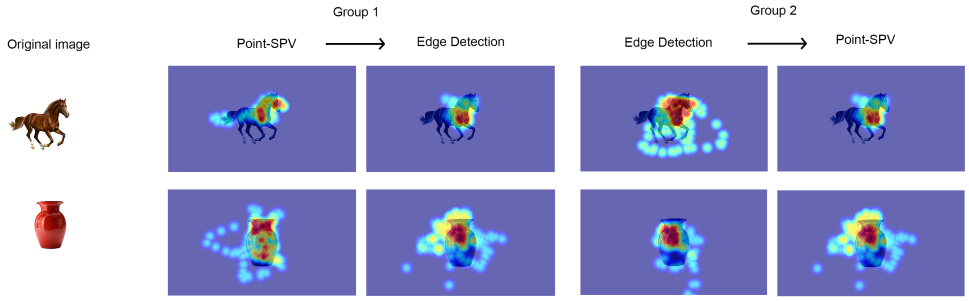

Figure S3. Average fixation heatmaps for two example stimuli across experimental conditions. Heatmaps are shown separately for Group 1 (switching from Point-SPV to Edge Detection) and Group 2 (switching from Edge Detection to Point-SPV), with visual attention averaged across all participants in each block. Warmer colors indicate areas with higher fixation density.

1.2 Reaction Time

Reaction times in the pilot experiment were comparable across the two methods, consistent with the high accuracy scores, suggesting that both representation techniques facilitated efficient recognition. Participants who began with Point-SPV responded, on average, in about 5 seconds, while those starting with edge detection had slightly longer reaction times, averaging around 6 seconds. After switching conditions, reaction times remained relatively stable with minimal variation, further indicating that both methods supported rapid task completion.

1.3 Number of Saccades

In terms of number of saccades, the pilot experiment showed minimal differences between Point-SPV and edge detection. Participants using Point-SPV exhibited an average of approximately 12 saccades per trial, while those starting with edge detection averaged slightly higher at around 14 saccades per trial. When participants switched between the two representation methods, number of saccades remained relatively consistent, with only a minor decrease. These modest differences, along with the high accuracy observed across both conditions, suggest that neither representation method required extensive visual exploration, likely due to the straightforward nature of the animate versus inanimate categorization task.

Interestingly, the participant with the longest reaction times also exhibited the highest number of saccades. However, this individual was not the same as the participant with the lowest accuracy. This finding implies that slower reaction times and higher saccade counts were not necessarily indicative of reduced recognition accuracy, but rather may reflect individual differences in visual processing strategies.

1.4 Stimulus Coverage

Participants who started with Point-SPV in the first block exhibited slightly lower average stimulus coverage, around 6%, compared to those beginning with edge detection, who covered approximately 7% of the stimulus surface. This indicates that Point-SPV directed participants' visual attention more efficiently, resulting in less extensive scanning behavior. When participants switched representation methods in the second block, stimulus coverage decreased for both groups, with a more pronounced reduction observed for those transitioning from edge detection to Point-SPV. These findings are consistent with the number of saccades data. Figure S3 illustrates examples of average coverage heatmaps across all participants for two sample stimuli.

1.5 Limitations and Exclusion

During the debriefing after the experiment, participants mentioned that distinguishing between animate and inanimate stimuli was relatively straightforward. They highlighted that the general shape of the target and the presence of straight edges around an object were strong indicators of the inanimate category. A potential ceiling effect can be observed in the results, leading us to exclude this experiment from the main body of the manuscript. However, this suggests that the representation method's efficiency may depend on the specific characteristics of the stimuli and the task.

2 INTERMEDIATE PIPELINES

In developing our methodology, we evaluated three different pipelines aimed at optimizing the model for object recognition using a high-resolution encoder. Our goal was to explore how variations in model architecture could influence the quality of visual representations.

The first pipeline adapted an existing end-to-end method based on an autoencoder (?). We enhanced this approach by integrating a classifier, enabling the model to jointly optimize visual representations using both cross-entropy loss and reconstruction loss. This modification aimed to improve object recognition by balancing accurate classification with reconstruction of input data.

For the second and third pipelines, we implemented our proposed model described in this paper, testing it with different configurations of VGGNet and ResNet152. These tests aimed to identify the most effective architecture for producing accurate and meaningful visual representations, particularly within the blind and sighted units.

Preliminary findings indicated that the ResNet152-based approach, used for both the blind and sighted units, outperformed the other pipelines. It generated the most visually meaningful representations, demonstrating its effectiveness in this context. Figure S4 shows a selection of random outputs from these intermediate pipelines, highlighting the performance differences among the three approaches.

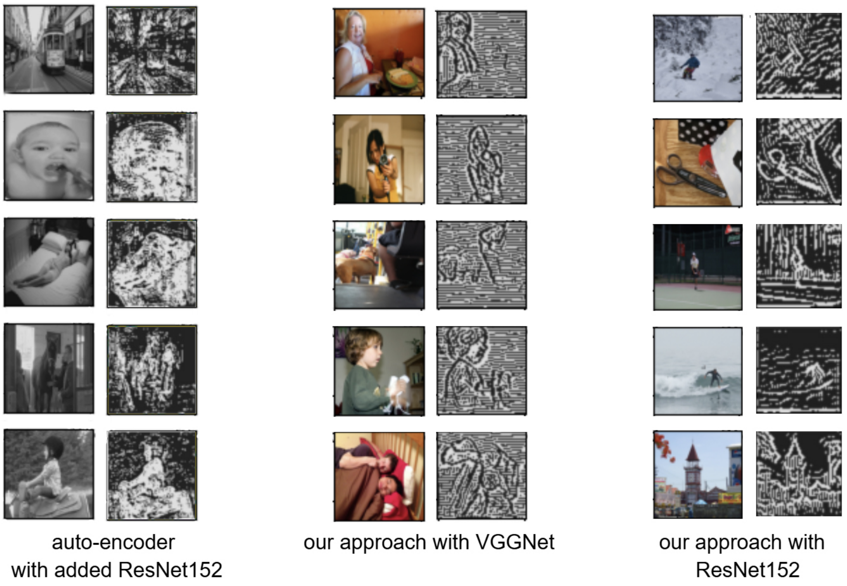

Figure S4. Random outputs from the encoders of three tested intermediate pipelines for this paper. The method on the right was chosen for further development based on potential in its visual representation.

3 TRAINING LOSS PROGRESS

As illustrated in Figure S5, the training process demonstrates a consistent decrease in both the validation and training loss values, as calculated using Equation **??**. This trend reflects the progressive learning of the model. Concurrently, the blind unit exhibits a steady improvement in recognition accuracy on the validation set, ultimately achieving an accuracy of approximately 73% on the validation set by the end of the training process.

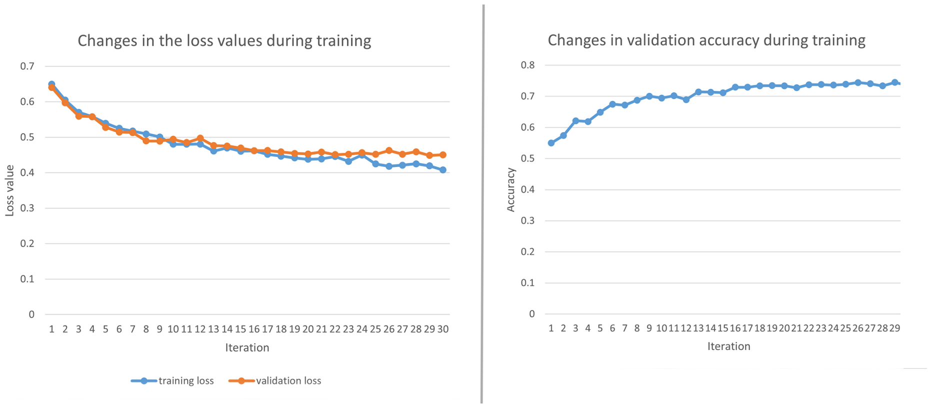

Figure S5. The left panel displays the progression of validation and training loss for the encoder, while the right panel illustrates the validation accuracy of the blind unit over 30 training iterations.
